# CRISPR/Cas9-mediated knockout of NSD1 suppresses the hepatocellular carcinoma development via the NSD1/H3/Wnt10b signaling pathway

**DOI:** 10.1186/s13046-019-1462-y

**Published:** 2019-11-14

**Authors:** Shuhua Zhang, Fan Zhang, Qing Chen, Chidan Wan, Jun Xiong, Jianqun Xu

**Affiliations:** 1 Department of Hepatobiliary Surgery of General Surgery, Union Hospital, Tongji Medical College, Huazhong University of Science and Technology, Wuhan, 430022 People’s Republic of China; 2 Department of Respiratory Medicine, Wuhan Third Hospital, Tongren Hospital of Wuhan University, Wuhan, 430060 People’s Republic of China

**Keywords:** NSD1, Wnt10b, Wnt/β-catenin signaling pathway, CRISPR/Cas9, Human hepatocellular carcinoma, Proliferation, Migration

## Abstract

**Background:**

The NSD family of histone lysine methyltransferases have emerged as important biomarkers that participate in a variety of malignancies. Recent evidence has indicated that somatic dysregulation of the nuclear receptor binding SET domain-containing protein 1 (NSD1) is associated with the tumorigenesis in HCC, suggesting that NSD1 may serve as a prognostic target for this malignant tumor. However, its mechanism in human hepatocellular carcinoma (HCC), the major primary malignant tumor in the human liver, remains unclear. Hence, we investigated how NSD1 regulated HCC progression via regulation of the Wnt/β-catenin signaling pathway.

**Methods:**

Reverse transcription quantitative polymerase chain reaction (RT-qPCR) and Western blot analysis was performed to identify the expression of NSD1 in HCC cells and clinically obtained tissues. The relationship between NSD1 expression and prognosis was analyzed by Kaplan-Meier survival curve. Further, a NSD1 knockout cell line was constructed by CRISPR/Cas9 genomic editing system, which was investigated in a battery of assays such as HCC cell proliferation, migration and invasion, followed by the investigation into NSD1 regulation on histone H3, Wnt10b and Wnt/β-catenin signaling pathway via ChIP. Finally, a nude mouse xenograft model was conducted in order to assess tumorigenesis affected by NSD1 knockout in vivo.

**Results:**

NSD1 was overexpressed in HCC tissues and cell lines in association with poor prognosis. Knockout of NSD1 inhibited the proliferation, migration and invasion abilities of HCC cells. CRISPR/Cas9-mediated knockout of NSD1 promoted methylation of H3K27me3 and reduced methylation of H3K36me2, which inhibited Wnt10b expression. The results thereby indicated an inactivation of the Wnt/β-catenin signaling pathway suppressed cell proliferation, migration and invasion in HCC. Moreover, these in vitro findings were reproduced in vivo on tumor xenograft in nude mice.

**Conclusion:**

In conclusion, the study provides evidence that CRISPR/Cas9-mediated NSD1 knockout suppresses HCC cell proliferation and migration via the NSD1/H3/Wnt10b signaling pathway, suggesting that NSD1, H3 and Wnt10b may serve as potential targets for HCC.

## Background

Human hepatocellular carcinoma (HCC), one of the most common malignancies, is recognized as the second leading cause of all-cancer related mortalities globally [1]. With a high mortality rate of 95% and an extremely short 5-year survival rate of 6.9%, HCC brings severe health burdens to patients’ families worldwide [2]. There are numerous risk factors closely associated with the alarming incidence of HCC including hepatitis B virus (HBV) and hepatitis C virus (HCV) infections, smoking, alcohol, dietary exposure to aflatoxins, diabetes, and obesity [3]. Despite advances in elucidating several mechanisms regulating the development and progression of HCC, such as the identification of critical tumor-related genes like TP53 and VEGFA, how the underlying molecular pathogenesis of HCC is driven through epigenetic programming remains poorly understood overall [4]. Given the precedent for epigenetic regulation in a wide range of human cancer, it is crucial to discover epigenetic molecular mechanisms of HCC cell proliferation and migration with the goal towards improving diagnosis and treatment for patients with HCC.

The nuclear receptor binding SET domain (NSD) proteins comprise a family composed of three methyltransferases including nuclear receptor binding SET domain-containing protein 1 (NSD1), NSD2 and NSD3, all of which are involved in tumorigenesis [5]. NSD1 is known to regulate gene expression through trimethylation of lysine 27 on histone H3 (H3K27me3) [6]. Previous evidence has revealed that the NSD methyltransferases are overexpressed in cancers, suggesting that these family members may serve as potential biomarkers for cancer diagnosis [7]. For example, NSD1/2 mutations in laryngeal tumors are closely associated with better prognosis [8]. In the context of HCC however, the regulatory mechanism of NSD1 remains largely unknown, with only a handful of studies showing a possible connection between NSD1 and liver disease [9]. Clustered regularly interspaced short palindromic repeats/CRISPR-associated 9 (CRISPR/Cas9) mediated genome-editing is an effective approach towards introducing mutations with a single sgRNA guide or generating knockout of genomic fragment with two or more sgRNA guides in human cells, which is widely applied to understand gene function and molecular mechanisms underlying diseases [10]. For instance, CRISPR/Cas9 induced mutations of p53 and Pten have been demonstrated to promote HCC development in HBV-transgenic mice [11]. Additionally, CRISPR/Cas9-mediated genome editing of CXC chemokine receptor 4 has been shown to suppress cell proliferation, migration and invasion of HCC in vitro and in vivo [12]. Separately, CRISPR-Cas9 mediated-NSD1 mutations in head and neck squamous cell carcinoma improve the survival of patients [13]. Wingless-related mouse mammary tumor virus integration site 10b (Wnt10b) is a member of the Wnt ligand gene family, which can activate the Wnt/β-catenin signaling pathway and plays a regulatory role in cell differentiation, proliferation and tumorigenesis in HCC [14, 15]. Promoting H3K27 methylation in the Wnt10b promoter represses its transcription and further suppresses the Wnt/β-catenin signaling pathway to promote adipogenesis [16]. However to date, no study has explored the connection underlying NSD1, H3K36 and Wnt10b in mediating the development of HCC, a void we aim to fill with this present investigation. This study attempts to provide theoretical support for the diagnosis and treatment of HCC by investigating the regulatory NSD1/H3/Wnt10b signaling pathway in HCC cell proliferation, migration and invasion.

## Materials and methods

### Ethics statement

Written informed consent was obtained from all patients prior to the study. Study protocols were approved by Ethic Committee of Union Hospital, Tongji Medical College, Huazhong University of Science and Technology and based on the ethical principles for medical research involving human subjects of the Helsinki Declaration. Animal experiments were conducted in strict accordance with the Guide to the Management and Use of Laboratory Animals issued by the National Institutes of Health. The protocol of animal experiments was approved by the Institutional Animal Care and Use Committee of Union Hospital, Tongji Medical College, Huazhong University of Science and Technology.

### Clinical samples

Sixty-three patients with HCC (mean age: 57.97 ± 3.45 years) who had received treatment at Union Hospital, Tongji Medical College, Huazhong University of Science and Technology from January 2013 to December 2015 were enrolled for the following assays. Through surgical excision avoiding the tumor necrosis and hemorrhage, samples including two copies of tumor and adjacent peritumoral tissues (3 cm away from the tumor), were obtained under sterile conditions. One sample was fixed in formalin solution, processed for tissue slicing, and then identified by two independent clinical pathologists using hematoxylin and eosin (HE) staining. The other sample was frozen in liquid nitrogen and then placed in a − 80 °C freezer for RNA and protein extraction. After discharge, patients were followed up until December 2018 for observing the 3-year survival status. The overall survival rate was calculated as the time from a random date to death [17].

### Cell culture and grouping

Human normal immortalized liver cell line (HL-7702) and HCC cell lines (Huh7, Hep3B, SMMC-7721, HepG2, and SK-Hep1) were obtained from American Type Culture Collection (ATCC) (https://www.atcc.org/), cultured in Dulbecco’s modified Eagle’s medium (DMEM) (Gibco, Carlsbad, CA, USA) supplemented with 10% fetal bovine serum (FBS, 10100147, Gibco BRL/Invitrogen, CA, USA) at 37 °C in an atmosphere of 5% CO_2_.

A lentivirus package system was designed with LV5-green fluorescent protein (GFP) (gene overexpression lentivirus vector) and pSIH1-H1-copGFP (gene silencing lentivirus short hairpin RNA [shRNA] vector with fluorescent expression). Wnt10b shRNA and its negative control shRNA (sh-NC) vectors were constructed by Shanghai GenePharma Co., Ltd. (Shanghai, China). The packaged virus and the shRNA vector were co-transfected into 293 T cells using Lipofectamine 2000. After 48 h of cell culture, the supernatant containing virus particles was collected through centrifugation. The titer of virus particles was determined by reverse transcription quantitative polymerase chain reaction (RT-qPCR). The virus in the exponential phase was then collected and treated with sh-NC, overexpressed (oe)-NC, sh-Wnt10b and oe-NSD1. HCC cells in the logarithmic phase were trypsinized, treated into single cell suspension of 5 × 10^4^ cells/mL, seeded into a 6-well plate (2 mL/well), and cultured at 37 °C overnight. The expression efficiency of GFP was observed under a fluorescence microscope at 48 h after treatment. The expression of related genes in each group of cells was determined by RT-qPCR. The experiment was run in triplicate [18].

### Cell model with NSD1 knockout using CRISPR-Cas9

Plasmid vectors including lentiCRISPRv2 (puro, catalog 52,961), psPAX2 (catalog 12,260) and pVSVg (catalog 8454) were used for NSD1 knockout in cells by CRISPR-Cas9. NSD1-specific single-guide RNA (sgRNA) was designed using the online tool CRISPR DESIGN (http://CRISPR.mit.edu), synthesized and cloned into lentiCRISPRv2. The sgRNA sequences are shown in Table 1. Lentiviruses were packaged in 293 T cells and then transfected into related cells. Monoclonal cell line was selected by puromycin. The cell line construction with knockout of NSD1 was verified by genomic sequencing and Western blot analysis [19].

**Table 1:** Primer sequences for NSD1-gRNA

Gene	Primer sequences
NSD1-gRNA1	F: 5′-TTGGATTGACCATTACCGAA-3′
NSD1-gRNA2	F: 5′-TGGATTGACCATTACCGAAA-3′
NSD1-gRNA3	F: 5′-GCAAGTGCTGTAGGACCACC-3′

Note: *F* Forward, *NSD1* Nuclear receptor binding SET domain protein 1

### Western blot analysis

The liver tissues or cells were lysed using radio-immunoprecipitation assay (RIPA) lysis buffer (20101ES60, Yeasen Biotech Co., Ltd., Shanghai, China) at 4 °C for 30 min and centrifuged for 15 min at 12000 g at 4 °C to collect the total protein. The protein concentration was determined using a bicinchoninic acid (BCA) protein quantification kit (Beyotime Institute of Biotechnology Co., Ltd., Shanghai, China). Then the protein was separated by 10% sodium dodecyl sulfate polyacrylamide gel electrophoresis and transferred on a polyvinylidene fluoride membrane (Millipore, Billerica, MA, USA) which was then sealed by 5% skimmed milk powder in Tris-buffered saline with Tween 20 (TBST) for 1 h. Next, the membrane was probed at 4 °C overnight with the following primary antibodies diluted by 5% milk TBST solution purchased from Abcam Inc., (Cambridge, MA, USA): mouse monoclonal antibody to NSD1 (ab70732, 1: 100), rabbit polyclonal antibody to Wnt10b (ab70816, 1: 100), rabbit polyclonal antibodies to H3K36me2 (ab9049, 1: 100) and H3K27me2 (ab24684, 1: 200), mouse monoclonal antibody to H3K27me3 (ab6002, 1: 100), rabbit polyclonal antibody to H3 (ab1791, 1: 1000), rabbit monoclonal antibodies to β-catenin (ab32572, 1: 5000), C-myc (ab32072, 1: 1000), CyclinD1 (ab16663, 1: 200) and glyceraldehyde-3-phosphate dehydrogenase (GAPDH) (ab181602, 1: 10000). The membrane was further incubated with horseradish peroxidase (HRP)-labeled secondary antibody (1: 5000, goat anti-mouse or rabbit, TransGen Biotech Co., Ltd., Beijing, China) at room temperature for 1 h. After that, the membrane was developed in enhanced chemiluminescence (JK30026.3, Shanghai Baoman Biotechnology Co., Ltd., Shanghai, China) and analyzed using Image J software, with GAPDH as an internal control. The experiment was run in triplicate.

### RNA isolation and quantitation

Total RNA was extracted from cells using Trizol (Invitrogen, Carlsbad, CA, USA). RNA quality and concentration were recorded using an ultraviolet-visible spectrophotometer (ND-1000, NanoDrop, Thermo Scientific, Wilmington, USA). RNA was reversely transcribed into complementary DNA (cDNA) by the PrimeScript RT reagent kit (Takara Biotechnology Co., Ltd., Dalian, Liaoning, China). Fluorescent qPCR was carried out in accordance with the instruction of SYBR® Premix Ex Taq™ II (Tli RNaseH Plus) Kit (TaKaRa Biotechnology Co., Ltd., Dalian, Liaoning, China). Primers were designed using the Primer Premier 5 software and then synthesized by Guangzhou RiboBio Co., Ltd. (Guangzhou, Guangdong, China) as shown in Table 2. GAPDH was used as an endogenous reference to normalize gene expression values with the 2^-ΔΔCt^ method. The experiment was run in triplicate.

**Table 2:** Primer sequences for RT-qPCR

Genes	Primer sequences
NSD1	F: 5′-AGGTGTAGAACACGATCCCG-3′
	R: 5′-AGCCGACCTTTAGATGCAGA-3’
Wnt10b	F: 5′-CACTGGAGGTCCTGATCGATC-3’
	R: 5′-CAGCCCCAAGGTAAGGCTGAC-3’
Wnt10b-promoter	F: 5′-TTTTGGATCCCAAGGCCCTC-3’
	R: 5′-GTTTGGCCCTAGCAGAGGTT-3’
GAPDH	F: 5′-GAAGGTGAAGGTCGGAGTC-3’
	R: 5′-GAAGATGGTGATGGGATTTC-3’

Note: *F* Forward, *R* Reverse, *RT-qPCR* Reverse transcription quantitative polymerase chain reaction, *NSD1* Nuclear receptor binding SET domain protein 1, *Wnt10b* Wingless-related mouse mammary tumor virus integration site 10b, *GAPDH* Glyceraldehyde-3-phosphate dehydrogenase

### Cell proliferation detection by cell counting kit-8 (CCK-8) method

The NSD1 knockout cells and normal control cells were taken for proliferation detection. After detachment, cells were counted with cell concentration adjusted, and seeded in a 96-well plate with 5000 cells per well. Then, 150 μL of medium was added in each well, followed by culture at 37 °C in a 5% CO_2_ incubator. After 24 h, 48 h, 72 h and 96 h of culture, the cells were incubated with 20 μL of CCK-8 reagent and 100 μL of medium in each well for 2 h devoid of exposure to light. The optical density value of each well was measured at 450 nm using a microplate reader as an indicator of cell growth viability and proliferation in a positive manner. Triplicate wells were set for each group.

### Monoclonal formation assay

The monoclonal formation assay was performed to detect the proliferation ability of tumor cells. In brief, the SK-Hep1 knockout cell line, the HepG2 overexpressing cell line and the control cell line in logarithmic phase were counted after detachment, and plated into a 6-well plate at a density of 1 × 10^3^ cells/well. After 7–10 days of culture, cell formation signaled the performance of crystal violet staining, and imaging was performed under a microscope. The experiment was run in triplicate.

### Cell migration and invasion detection by Transwell assay

Detection of cell migration and invasion in vitro was performed in a 24-well plate using a Transwell chamber (pore size: 8 μm; Corning, NY, USA). DMEM (600 μL) containing 20% FBS was pre-added to the Transwell chamber of polycarbonate membrane coated with matrigel and the matrigel-free Transwell chamber, respectively, and equilibrated at 37 °C for 1 h. HCC cells after 48 h of transfection were resuspended in DMEM containing 10% FBS, added to the upper chamber at a density of 1 × 10^9^ cells/100 μL, and then cultured at 37 °C in a 5% CO_2_ atmosphere for 24 h. Cells in the inner layer of the Transwell microporous membrane were removed by swab. Following this, cells on the outer layer of the Transwell microporous membrane were fixed with 4% methanol, and stained with 0.1% crystal violet solution. The stained cells were subsequently counted under an inverted microscope and then photographed. Then 5 fields of view were randomly selected. The experiment was run in triplicate.

### Chromatin immunoprecipitation (ChIP) assay

ChIP assay was performed using the EZ-Magna ChIP TMA Kit (Millipore, Billerica, MA, USA). HCC cells and NSD1 knockout HCC cells in logarithmic growth phase were cross-linked with 1% polyformaldehyde solution for 10 min, and the reaction was stopped by reacting with 125 mM glycine solution at room temperature for 5 min. The cells were then washed twice with pre-cooled phosphate buffer saline (PBS), centrifuged for 5 min at 2000 rpm, and resuspended in a cell lysis [150 mM NaCl, 50 mM Tris (pH 7.5), 5 mM ethylenediaminetetraacetic acid (EDTA), 0.005% NP40, 0.01% Triton X-100) for a final concentration of 2 × 10^6^ cells/200 mL. Cells were added with the protease inhibitor cocktail (PIC) mixture, centrifuged at 5000 rpm for 5 min, resuspended in nuclear separation buffer, lysed in an ice water bath for 10 min, and ultrasonically cleaved to obtain chromatin fragments (200–1000 bp). The supernatant was extracted by centrifugation at 14000 g for 10 min at 4 °C. A total amount of 100 μL supernatant (DNA fragment) was added to 900 μL of ChIP Dilution Buffer and 20 μL of 50 × PIC, followed by addition of 60 μL Protein A Agarose/Salmon Sperm DNA. After mixing at 4 °C for 1 h, the cells were allowed to stand at 4 °C for 10 min and centrifuged at 700 rpm for 1 min. The 20 μL supernatant was taken as the input. The supernatant was incubated with 1 μL rabbit antibody against H3K27me3 (ab6002, 1: 100, Mouse) overnight in the experimental group, and with 1 μL of rabbit antibody against Immunoglobulin G (IgG) (ab172730) in the NC group. All antibodies were purchased from Abcam Inc., (Cambridge, MA, USA). Each tube was added with 60 μL Protein A Agarose/Salmon Sperm DNA, and then inverted at 4 °C for 2 h. After the tubes stood for 10 min, centrifugation was performed at 700 rpm for 1 min. The supernatant was removed and the precipitate was washed with 1 mL low salt buffer, high salt buffer, LiCl solution, and twice with Tris-EDTA buffer solution, respectively. Each tube was eluted twice with 250 mL ChIP Wash Buffer, and then decrosslinked by 20 mL of 5 M NaCl, followed by the recovery of DNA. The promoter of Wnt10b DNA was quantified by fluorescence qPCR. The Wnt10b promoter primer sequences are shown in Table 1.

### Xenograft tumor in nude mice

A total of 60 male BALB/c nude mice (age: 3–5 weeks, weight: 18–22 g) were purchased from Shanghai Experimental Animal Center of Chinese Academy of Sciences (Shanghai, China), and housed in specific pathogen free laboratory at 24–26 °C under constant humidity of 40–60% with access to autoclaved standard laboratory feed and sterile drinking water. Mice were exposed to ultraviolet radiation on a regular basis. The cells treated with CRISPR-Cas9 vector, NSD1-KO, sh-NC, sh-Wnt10b, NSD1-KO + sh-Wnt10b (Shanghai GenePharma Co., Ltd., Shanghai, China) in logarithmic phase were dispersed into cell suspension with concentration adjusted to 5 × 10^6^ cells/mL with PBS solution. After 1 week, 200 μL cell suspension (1 × 10^6^ cells per 0.2 mL) was subcutaneously injected into male BALB/cA-nu nude mice (*n* = 12). One week after the injection, the short diameter (a) and the long diameter (b) of the tumors were recorded with a caliper, and the tumor volume (V) was calculated by the formula: V = π (a^2^ × b)/6. The tumor mass was weighed by the balance, and the measurement was repeated 3 times for each group. Tumor tissues from sacrificed mice were removed at 28th day post inoculation, fixed in 4% polyformaldehyde solution, dehydrated, embedded in paraffin and cut into 4 μm slices. Tumor tissue RNA was extracted to determine the expression of β-catenin, C-myc and CyclinD1 [20].

### Immunohistochemical staining

Immunohistochemical staining was performed using streptavidin-perosidase (SP) method. Antigen retrieval was conducted by boiling the slices in a microwave oven (twice at an interval of 5 min) and cooled down to room temperature. The slices were blocked by a normal goat serum. The vector and the sh-NC group were used as NC. A HistostainTM SP-9000 immunohistochemical staining kit (Zymed Laboratories, San Francisco, CA, USA) was used for staining. The slices were then probed with primary rabbit monoclonal antibody to β-catenin (ab32572, 1: 5000), rabbit monoclonal antibody to C-myc (ab32072, 1: 1000), and rabbit monoclonal antibody to CyclinD1 (ab16663, 1: 200) at 4 °C overnight. All antibodies were purchased from Abcam Inc. (Cambridge, MA, USA). The slices were then incubated with secondary goat anti-mouse or goat anti-rabbit (TransGen Biotech Co., Ltd., Beijing, China) at 37 °C for 30 min. After that, the slices were further incubated with HRP-labeled solution, and developed with diaminobenzidine for 5–10 min. After counterstaining by hematoxylin for 1 min, the slices were sealed with gum, and photographed under an upright optical microscope (NIKON, Tokyo, Japan). Five representative high-power fields were randomly selected. The cells with brown or yellow cytoplasm were regarded positive.

### TOPFlash luciferase assay

Exponentially growing NSD1 knockout cells were seeded in 100 mm cell culture dishes and treated with Ad-TOP-Luc reporter virus for approximately 16 h, and then re-plated into 24-well plates, followed by infection with different sh-NC or sh-Wnt10b. At 24 h and 36 h after treatment, the cells were lysed and subjected to luciferase activity assays using the Firefly Luciferase Assay System (Promega, Madison, WI, USA). The experiment was run in triplicate [21].

### Statistical analysis

Statistical analysis was conducted by SPSS 21.0 (IBM Corp. Armonk, NY, USA). Measurement data were expressed as mean ± standard deviation (s.d.). Differences between HCC tissues and adjacent normal tissues were compared by paired *t* test while other two groups were compared by independent sample *t* test. Data among multiple groups were analyzed by one-way analysis of variance (ANOVA), followed by Tukey’s post-hoc test. Data at different time points were analyzed by repeated measures ANOVA, followed by Bonferroni’s post-hoc test. Kaplan-Meier method (log-rank test) was used to analyze the relationship between NSD1 expression in HCC tissues and overall survival of patients with HCC. *p* < 0.05 was used as the threshold for statistical significance.

## Results

### NSD1 is overexpressed in HCC tissues and cells

**Fig. 1:**
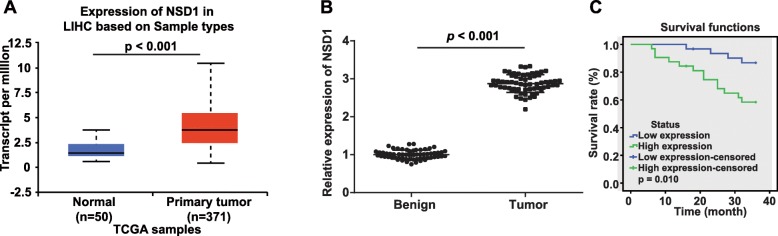
High expression of NSD1 in HCC was associated with poor prognosis. **a**, Expression of NSD1 in normal tissue and primary HCC tumor tissue analyzed by microarray-based analysis (http://ualcan.path.uab.edu/index.html). **b**, Relative expression of NSD1 in adjacent normal and HCC tissues (*n* = 63) determined by RT-qPCR. **c**, Kaplan-Meier survival curve for analysis on the relation between NSD1 expression and prognosis in patients with HCC. * *p* < 0.05 vs. adjacent normal tissues. Data (mean ± s.d.) between two groups were examined by paired *t* test

To identify candidate genes associated with HCC, we first undertook a microarray-based gene expression analysis (http://ualcan.path.uab.edu/index.html). Our results revealed that NSD1 was overexpressed in HCC (Fig. 1a). To build on this initial finding, we queried the expression of NSD1 in clinically obtained HCC tissues along with adjacent normal controls by RT-qPCR. Encouragingly, we once again found NSD1 expression to be significantly higher in HCC tissues over adjacent normal tissues (Fig. 1b). To assess whether this higher expression of NSD1 can account for overall survival in patients with HCC, we first stratified patients into two groups: ones possessing high NSD1 expression (*n* = 32) or low NSD1 expression (*n* = 31) based on the median NSD1 expression value (*n* = 2.862). We then assessed survival by a Kaplan-Meier survival curve, and were able to further confirm that patients with NSD1 overexpression did indeed go on to show poor prognosis (Fig. 1c). Taken together, these aforementioned results demonstrate that NSD1 is overexpressed in HCC, and this overexpression is closely associated with poor prognosis.

### NSD1 knockout inhibits the proliferation, migration and invasion abilities of HCC cells

**Fig. 2:**
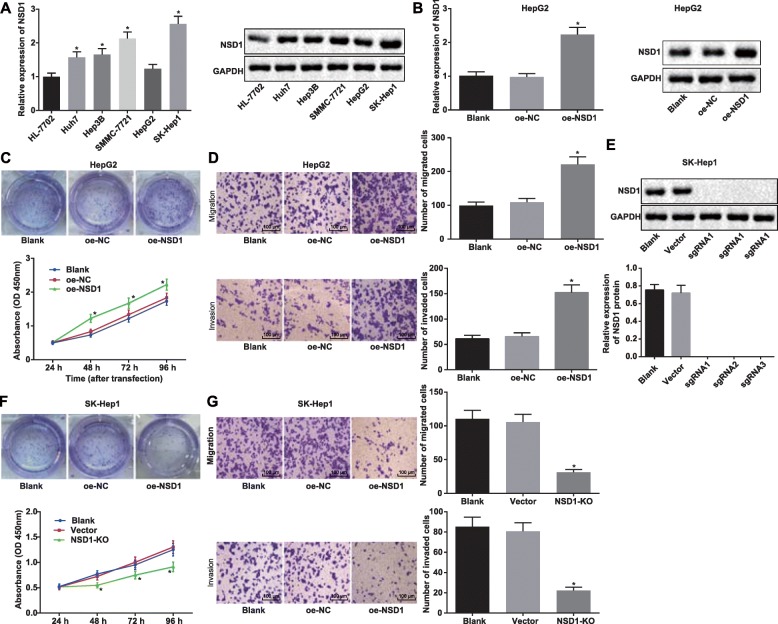
NSD1 knockout inhibits cell proliferation, migration and invasion abilities in HCC. **a**, Expression of NSD1 in human immortalized liver cell line (HL-7702) and HCC cell lines (Huh7, Hep3B, SMMC-7721, HepG2, and SK-Hep1) determined by RT-qPCR and Western blot analysis. **b**, mRNA and protein expression following NSD1 overexpression in HepG2 cell line examined by RT-qPCR and Western blot analysis. **c**, Proliferation ability of HepG2 cells after overexpression of NSD1 determined by CCK-8 method and monoclonal formation assay. **d**, Migration and invasion ability of HepG2 cells following NSD1 overexpression determined by Transwell assay (× 100). **e**, Expression of NSD1 normalized to GAPDH in SK-Hep1 cells after knockout of NSD1 by CRISPR-Cas9 examined by Western blot analysis. **f**, Proliferation ability of SK-Hep1 cells after knockout of NSD1 measured by CCK-8 method and monoclonal formation assay. **g**, Migration and invasion ability of SK-Hep1 cells after knockout of NSD1 detected by Transwell assay (× 100). * *p* < 0.05 vs. the HL-7702 cell line, or the blank group (HepG2/SK-Hep1 cells without any treatment). Data (mean ± s.d.) among multiple groups were analyzed by one-way ANOVA, followed by Tukey’s post-hoc test, while those at different time points were analyzed by repeated measures ANOVA, followed by the Bonferroni’s post-hoc test. The experiment was repeated 3 times independently

To explore NSD1 expression across cell lines, we first collated a list of HCC cell lines and then assessed expression via RT-qPCR and Western blot analysis. We chose the human immortalized liver cell line (HL-7702) and several HCC cell lines (Huh7, Hep3B, SMMC-7721, HepG2, and SK-Hep1) for this analysis, and found that NSD1 had the highest expression in SK-Hep1 cell line and the lowest expression in HepG2 cell line, suggesting that expression of NSD1 in HCC cell lines was significantly higher than that in the normal cell line (Fig. 2a). We therefore picked SK-Hep1 and HepG2 cell lines for further experiments going forward. We initially wanted to explore whether NSD1 regulates the proliferation and the migration ability of HCC cells, and began our exploration by overexpressing NSD1 in the chosen cell lines. We first validated the expression efficiency of transduction by RT-qPCR and Western blot analysis and indeed confirmed higher NSD1 expression after transduction of overexpressed NSD1 vector (Fig. 2b). To assess cellular proliferation, we set up the CCK-8 and monoclonal formation assays, which clearly demonstrated that overexpressed NSD1 enhanced the cellular proliferation ability in HepG2 cell line (Fig. 2c). Next, the transwell assay was used to analyze the cellular migration and invasion ability of HepG2 cells upon NSD1 overexpression, and once again we observed enhanced cellular invasion and migration phenotypes (Fig. 2d). As an orthogonal approach, we also created CRISPR-Cas9 mediated NSD1 knockouts (Fig. 2e), and demonstrated via CCK-8 and monoclonal formation assays that NSD1 knockout reduces the proliferation ability of cells. Transwell assays also showed that knockout of NSD1 inhibited cellular migration and invasion ability (Fig. 2f). Collectively, the above data illustrate that NSD1 levels correlate with proliferation, migration and invasion ability of HCC cells.

### NSD1 promotes Wnt10b transcription by inhibiting H3K27me3 methylation in the Wnt10b promoter region

**Fig. 3:**
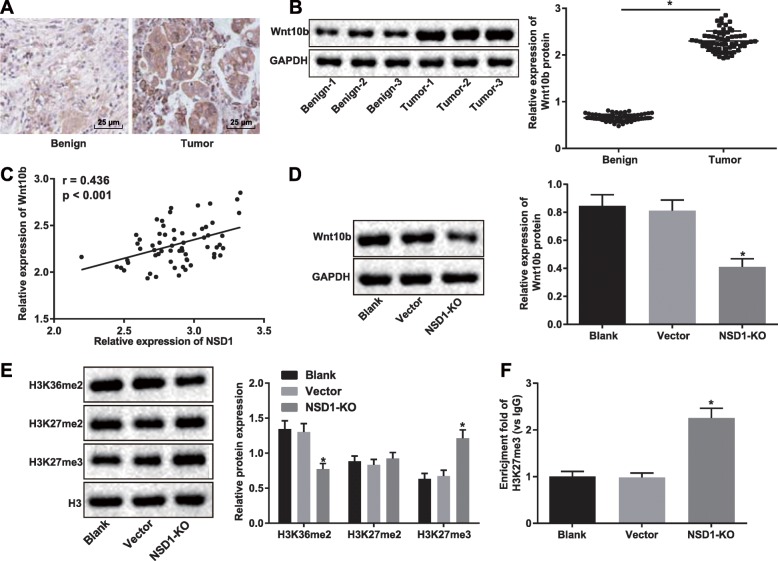
NSD1 regulates H3K27me3 methylation in the Wnt10b promoter region to promote Wnt10B transcription. **a**, Wnt10b expression in adjacent normal and HCC tissues identified by immunohistochemical staining (× 400). **b**, Protein expression of Wnt10b normalized to GAPDH in adjacent normal and HCC tissues (*n* = 63) determined by Western blot analysis. **c**, Correlation analysis between expression of NSD1 and Wnt10b in HCC. **d**, Protein expression of Wnt10b normalized to GAPDH in HCC cells following NSD1 knockout determined by Western blot analysis. **e**, Protein expression of H3K36me2, H3K27me2, H3K27me3 in cells after knockout of NSD1 determined by Western blot analysis. **f**, Enrichment of H3K27me3 in Wnt10b promoter region in cells after knockout of NSD1 detected by ChIP assay. * *p* < 0.05 vs. adjacent normal tissues, or the blank group (HepG2/SK-Hep1 cells without any treatment). Data (mean ± s.d.) between two groups were analyzed by paired *t* test, and those among multiple groups were analyzed by one-way ANOVA, followed by Tukey’s post-hoc test, while data at different time points were analyzed by repeated measures ANOVA, followed by the Bonferroni’s post-hoc test. The experiment was repeated 3 times independently

Wnt10b has previously been reported to be overexpressed in HCC [22]. We first performed immunohistochemical staining and Western blot analysis to determine Wnt10b expression in HCC and adjacent normal tissues, and showed that Wnt10b was significantly overexpressed in HCC tumor tissue (Fig. 3a-b). Curious whether there was a correlation between NSD1 and Wnt10b, we examined the expression of both NSD1 and Wnt10b in HCC patients, and indeed discovered a positive correlation between NSD1 expression and Wnt10b expression from our correlation analysis (Fig. 3c). Next, we determined that NSD1 knockout was sufficient to inhibit the expression of Wnt10b, as assessed by Western blot analysis (Fig. 3d). The methyltransferase enzyme EZH2 is known to regulate the enrichment of H3K27me3 in PRC2 target genes (Wnt1, Wnt6, Wnt10a and Wnt10b) to promote adipogenesis [23]. Enrichment of H3K27me3 in the Wnt10b promoter region is also known to inhibit the Wnt/β-catenin signaling pathway [16]. Therefore, to further explore the modular mechanism between NSD1 and Wnt10b, we examined the expression of H3K36me2, H3K27me2, H3K27me3 and H3 in response to knockout of NSD1 in cells by Western blot analysis. We found H3K27me3 expression to be notably increased (Fig. 3e), and ChIP assay also confirmed an attenuated enrichment of H3K27me3 in the Wnt10b promoter region in SK-Hep1 cells after knockout of NSD1 compared to wild-type cells (Fig. 3f). The above results reveal that knockout of NSD1 in HCC cells promotes the enrichment of H3K27me3 in Wnt10b promoter region, thereby reducing the expression of Wnt10b.

### CRISPR/Cas9-mediated knockout of NSD1 inhibits the Wnt/β-catenin signaling pathway to suppress HCC cell proliferation, migration and invasion

**Fig. 4:**
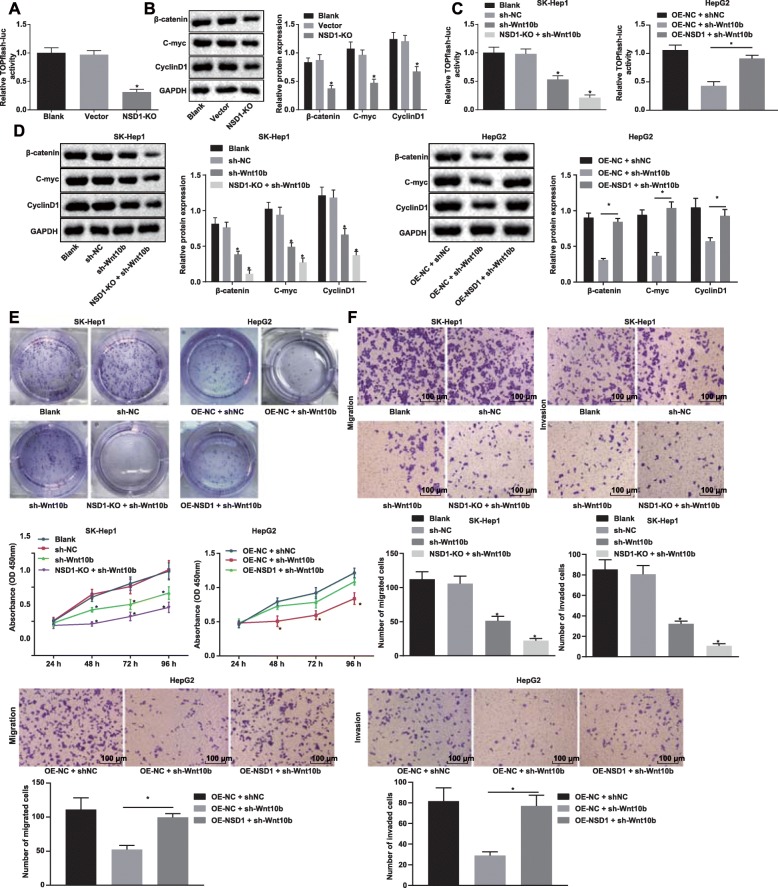
Knockout of NSD1 inhibits the Wnt/β-catenin signaling pathway to suppress HCC cell proliferation, migration and invasion. **a**, Relative TOPFlash luciferase activity after NSD1 knockout. **b**, Protein expression of key proteins β-catenin, C-myc and CyclinD1 in the Wnt/β-catenin signaling pathway normalized to GAPDH after Wnt10b knockout determined by Western blot analysis. **c**, Relative TOPFlash luciferase activity after Wnt10b silencing, NSD1 silencing or both NSD1 overexpression and Wnt10b silencing. **d**, Protein expression of Wnt10b and key proteins, β-catenin, C-myc and CyclinD1 in the Wnt/β-catenin signaling pathway normalized to GAPDH determined by Western blot analysis. **e**, Proliferation ability of cells in different groups measured by CCK-8 and monoclonal formation assays. **f**, Migration and invasion ability of cells in differently groups detected by Transwell assay (× 100). * *p* < 0.05 vs. the blank group (SK-Hep1/HepG2 cells without any treatment). Data (mean ± s.d.) among multiple groups were analyzed by one-way ANOVA, followed by Tukey’s post-hoc test, while data at different time points were analyzed by repeated measures ANOVA, followed by the Bonferroni’s post-hoc test. The experiment was repeated 3 times independently

To further explore the mechanism of NSD1 regulating Wnt10b in HCC, we used the TOPFlash luciferase assay to examine the activity of the Wnt/β-catenin signaling pathway in cells after stable knockout of NSD1 by CRISPR/Cas9. Our results showed that knockout of NSD1 inhibited the activity of the Wnt/β-catenin signaling pathway in cells (Fig. 4a). Next, as assessed by Western blot analysis, we observed a significant reduction in β-catenin, C-myc and CyclinD1, which are key proteins of Wnt/β-catenin signaling pathway, following NSD1 knockout in cells (Fig. 4b).

We next, subjected HepG2 cells to harboring either overexpressed NSD1 or knocked-out NSD1 to shRNA mediated Wnt10b silencing. TOPFlash luciferase assay for the activity of the Wnt/β-catenin signaling pathway showed that silencing Wnt10b inhibited the activity of the Wnt/β-catenin signaling pathway and the NSD1 knockout further reduced the activity of the Wnt/β-catenin signaling pathway in these cells. When Wnt10b was knocked down in cells overexpressing NSD1, the effects of both were neutralized (Fig. 4c). Western blot analysis for the expression determination of the Wnt/β-catenin signaling pathway related key proteins, β-catenin, C-myc and CyclinD1, revealed similar trends as results of the TOPFlash luciferase assay (Fig. 4d). To further explore the mechanism by which NSD1 regulates the biological functions of Wnt10b, CCK-8 and monoclonal formation assays were used to examine the proliferation capacity of cells. We observed the proliferation ability of cells to be significantly reduced upon Wnt10b silencing, and this reduction was further enhanced in an NSD1 knockout setting. Once again, overexpression of NSD1 was sufficient to reverse the reduction in proliferation from Wnt10b silencing alone (Fig. 4e). Finally, silencing Wnt10b also significantly inhibited cell migration and invasion ability, both of which were further reduced upon NSD1 knockout. Inhibitory effects of Wnt10b or NSD1 silencing, as mentioned above, were partially abolished by both NSD1 overexpression and Wnt10b silencing (Fig. 4f). Taken together, these results verified that knockout of NSD1 inhibits HCC cell proliferation, migration and invasion by silencing Wnt10b via inactivating the Wnt/β-catenin signaling pathway.

### Knockout of NSD1 inhibits tumor formation and metastasis through downregulation of Wnt10b by inactivating the Wnt/β-catenin signaling pathway in nude mice

**Fig. 5:**
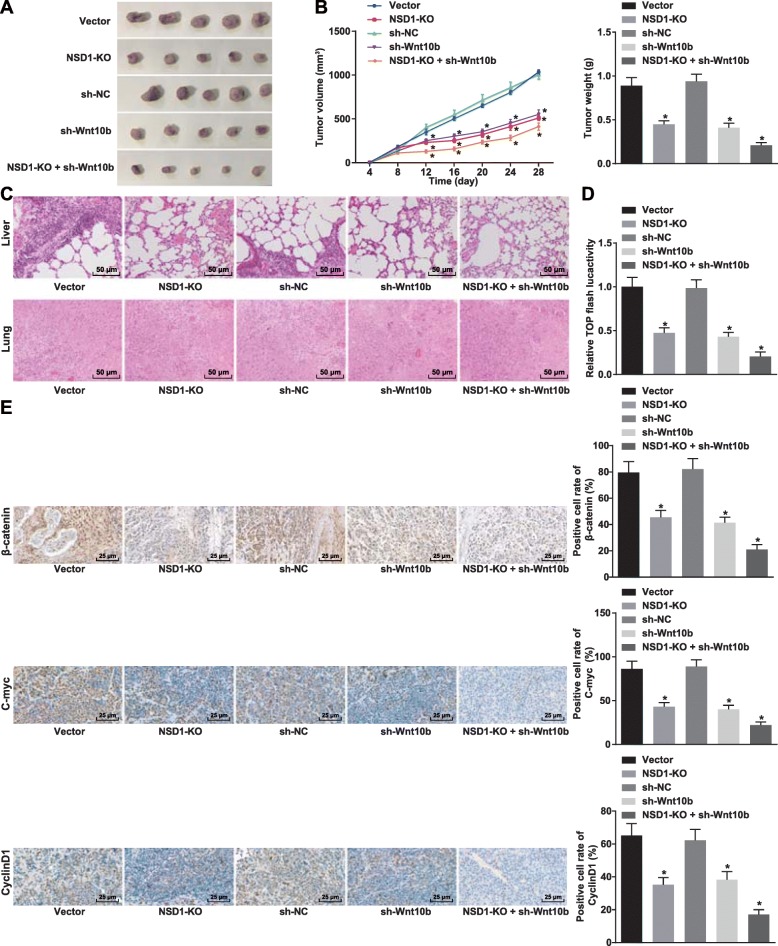
Silencing Wnt10b after knockout of NSD1 inhibits the formation and metastasis of tumors by inactivating the Wnt/β-catenin signaling pathway. **a**, Representative images of tumor formation through nude mouse xenograft model. **b**, Volume and weight of tumors. **c**, Pulmonary metastasis (× 200) detected by HE staining. **d**, TOPFlash luciferase activity in vivo after NSD1 knockout and silencing Wnt10b. **e**, Positive cell rates of β-catenin, C-myc, and CyclinD1 in tumors (× 400) detected by immunohistochemical staining. * *p* < 0.05 vs. the vector group (nude mice treated with vector). Data (mean ± s.d.) among multiple groups were analyzed by one-way ANOVA, followed by Tukey’s post-hoc test, while data at different time points were analyzed by repeated measures ANOVA, followed by the Bonferroni’s post-hoc test, *n* = 12

Nude mouse xenograft model was established to validate the functional consequences of NSD1 knockout. Cell lines harboring silenced Wnt10b and silenced Wnt10b together with knockout of NSD1 were injected to nude mice to assess the volume and weight of xenograft tumors. Intriguingly, we found that the volume and weight of the xenograft tumors in nude mice were significantly reduced upon NSD1 knockout or Wnt10b silencing. Strikingly, silencing Wnt10b in combination with a knockout of NSD1, further suppressed the volume and weight of xenograft tumor in nude mice (Fig. 5a-b). Next, HE staining was used to examine pulmonary metastasis in nude mice after injected with transfected cells for 28 days. Once again we found that while pulmonary metastasis was suppressed after knockout of NSD1 or silencing Wnt10b, the combination of the two further suppressed pulmonary metastasis significantly (Fig. 5c). We then examined the activity of Wnt/β-catenin signaling pathway in each group of tumors by means of the TOPFlash luciferase assay, and confirmed that knockout of NSD1 or silencing Wnt10b decreased Wnt/β-catenin signaling pathway activity, and that silencing Wnt10b after knockout of NSD1 further reduced Wnt/β-catenin signaling pathway activity over the individual genetic perturbations alone (Fig. 5d). Immunohistochemical staining of key Wnt/β-catenin signaling pathway proteins β-catenin, C-myc and CyclinD1 revealed a reduction in percentage positive cells for all markers after knockout of NSD1 or silencing Wnt10b alone and a stronger reduction upon combination of Wnt10b silencing and NSD1 knockout (Fig. 5e). Collectively these results demonstrate that silencing Wnt10b in combination with NSD1 knockout imposes a significant blockade in the Wnt/β-catenin signaling pathway, and thereby strongly inhibits tumor formation as well as extent of metastatic lesions in vivo.

## Discussion

HCC is the one of common malignant cancers, that can be attributed to HBV, HCV, alcohol abuse and other risk factors [24]. HCC is typically characterized with a low survival rate, and therefore merits the execution of early measures to prevent the onset of this cancer type [25]. It is therefore imperative to identify novel HCC-related molecules for the discovery of new prognostic markers and therapeutic targets. Despite the understanding on several factors responsible for the occurrence of this solid cancer, the underlying molecular mechanisms of HCC still await further investigation [26]. The NSD family of histone lysine methyltransferases has been reported to be overexpressed in multiple malignancies [7]. The somatic dysregulation of NSD1 is associated with tumorigenesis [27], suggesting that NSD1 may serve as a prognostic target for the treatment of cancers. With this in mind, we were interested in studying the regulatory role of NSD1 in HCC by genetic knockout approaches. Collectively, our data from this study shows that CRISPR/Cas9-mediated gene knockout of NSD1 can inhibit HCC cell proliferation, migration and invasion both in vitro and in vivo through H3K27me3 enrichment and Wnt10b downregulation.

We first identified that the expression of NSD1 is significantly higher in HCC tissues and cell lines, and that NSD1 levels regulate proliferation, migration and invasion of HCC cells. Previous evidence has shown that NSD1 belongs to a family of mammalian histone lysine methyltransferases (NSD1, NSD2, and NSD3) that play a regulatory role in multiple aspects of development and disease [28]. Hypermethylation exerted by NSD1 is also associated with poor outcomes in high-risk neuroblastoma [29]. NSD1 gene fusion is strongly associated with poor prognosis in pediatric acute myeloid leukemia [30]. Evidence of any break of NSD1 gene or reduction of its expression suggests good prognosis. For example, silencing NSD1 promotes cell apoptosis in association with inflammation [31]. Inactivating NSD1 deregulates DNA methylation in head and neck squamous cell carcinoma [32]. The CRISPR/Cas9 system has emerged as a highly efficient and powerful tool for RNA-guided editing of the cellular genome, and is wildly implemented to drill down on functions of genes [10]. As examples from the HCC literature, CRISPR-Cas9 mediated knockout of Nogo-B was shown to restrain HCC cell proliferation, migration and invasion [33], and in a separate study, CRISPR/Cas9 mediated knockout of eEF2 kinase decreases cell proliferation and growth in HCC [34]. A previous report also demonstrated that CRISPR-Cas9 mediated NSD1 mutations in head and neck squamous cell carcinoma lead to an improvement in the survival of patients [13]. These results are strongly consistent with the findings in this study that CRISPR/Cas9-mediated knockout of NSD1 can indeed inhibit cell proliferation, migration and invasion of HCC cells.

Subsequently, we found that NSD1 inhibits H3K27me3 methylation and promotes Wnt10b transcription. Methylation of H3 may happen in multiple sites of H3, including K27 and K36 which arises and falls of the other. It has been proven that the loss of NSD1-mediated generation of H3K36me2 attributes to the accumulation of H3K27me3 in embryonic stem cells [6]. Overexpressed NSD2 in myeloma cells is known to significantly increase the level of H3K36 dimethylation, followed by a striking decrease in H3K27 methylation [35]. In recent years, hypermethylation-mediated regulation of tumor suppressor genes and oncogenes are well-known regulatory models for the aberrant epigenetic modifications [36]. High expression of H3K27me3 is reported to be associated with larger tumor size, vascular invasion, poor differentiation and unfavorable prognosis in patients with HCC [37]. Promoting H3K27 methylation decreases the expression of Wnt10b and further suppresses the Wnt/β-catenin signaling pathway to promote adipogenesis [16]. As a member of the Wnt gene family, Wnt10b gene is overexpressed in HCC tissues and cells, silencing of which significantly reduces cell proliferation, migration, invasion, and colony formation in HCC [22]. Wnt10b can activate the Wnt/β-catenin signaling pathway and plays a role in cell proliferation and tumorigenesis in HCC [38]. The above-mentioned results are in full support of our findings that knockout of NSD1 promotes H3K27me3 methylation and inhibits Wnt10b transcription, thereby suppressing activation of the Wnt/β-catenin signaling pathway. Finally, using a xenograft nude mouse model we demonstrated that knockout of NSD1 suppresses the expression of Wnt10b, thus inhibiting the proliferation and migration of tumor cells in vivo by inactivating the Wnt/β-catenin signaling pathway. However, how Wnt10b affects the cancer cellular physiology by NSD1 alteration should be the subject of further investigation.

## Conclusion

**Fig. 6:**
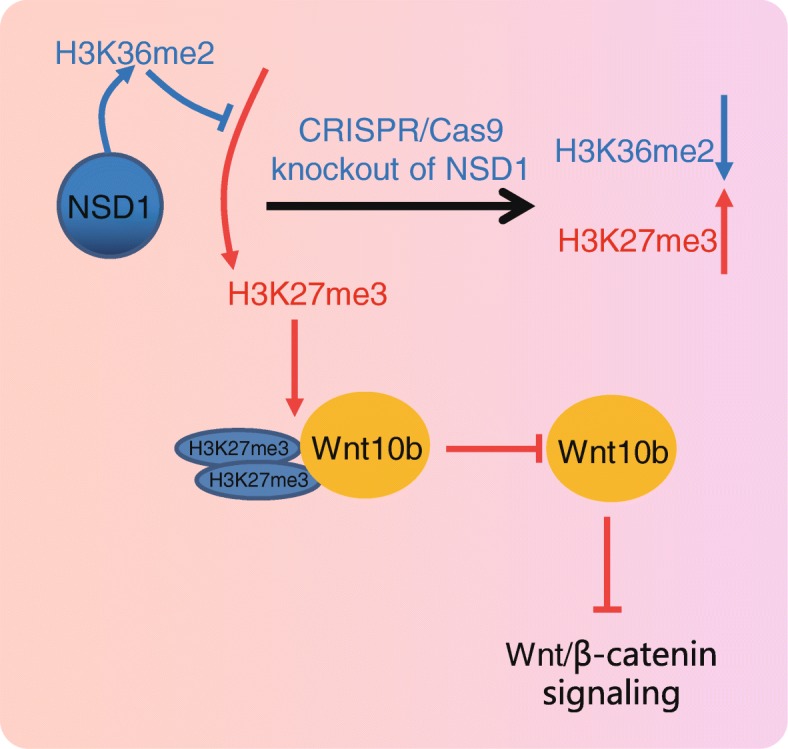
The mechanism scheme uncovers that CRISPR/Cas9-mediated knockout of NSD1 promotes H3K27me3 methylation and inhibits Wnt10b transcription, thereby suppressing activation of the Wnt/β-catenin signaling pathway

In conclusion, available evidence in our study elucidates that NSD1 is highly expressed in HCC cells in relation to poor prognosis in patients with HCC. Knockout of NSD1 mediated by CRISPR/Cas9 system promotes H3K27me3 methylation and inhibits Wnt10b transcription, thereby suppressing activation of the Wnt/β-catenin signaling pathway (Fig. 6), which finally impedes cell proliferation, migration and invasion in HCC. These findings demonstrate that NSD1 may serve as a promising diagnostic biomarker for HCC. At present, the regulatory mechanism of CRISPR/Cas9 mediated knockout of NSD1 with H3K27me3/Wnt10b remains scantly identified in HCC, and we will further study the underlying rules governing NSD1/H3/Wnt10b interaction in future studies.

## Data Availability

The datasets generated/analyzed during the current study are available.

## Abbreviation

ANOVA: Analysis of variance
ATCC: American Type Culture Collection
BCA: Bicinchoninic acid
CCK-8: Cell counting kit-8
ChIP: Chromatin immunoprecipitation
CRISPR/Cas9: Clustered regularly interspaced short palindromic repeats/CRISPR-associated 9
DMEM: Dulbecco’s modified Eagle’s medium
EDTA: Ethylenediaminetetraacetic acid
FBS: Fetal bovine serum
GAPDH: Glyceraldehyde-3-phosphate dehydrogenase
GFP: Green fluorescent protein
H3K27me3: Trimethylation of lysine 27 on histone H3
HBV: Hepatitis B virus
HCC: Hepatocellular carcinoma
HCV: Hepatitis C virus
HE: Hematoxylin and eosin
HRP: Horseradish peroxidase
IgG: Immunoglobulin G
NC: Negative control
NSD: Nuclear receptor binding SET domain
NSD1: Nuclear receptor binding SET domain-containing protein 1
oe: overexpressed
PBS: Phosphate buffer saline
PIC: Protease inhibitor cocktail
RIPA: Radio-immunoprecipitation assay
RT-qPCR: Reverse transcription quantitative polymerase chain reaction
sgRNA: single-guide RNA
shRNA: short hairpin RNA
SP: Streptavidin-perosidase
TBST: Tris-buffered saline with Tween 20
UTR: Untranslated region
Wnt10b: Wingless-related mouse mammary tumor virus integration site 10b
